# Characterization of A Bifunctional Synthetic RNA Aptamer and A Truncated Form for Ability to Inhibit Growth of Non-Small Cell Lung Cancer

**DOI:** 10.1038/s41598-019-55280-x

**Published:** 2019-12-11

**Authors:** Hanlu Wang, Meng Qin, Rihe Liu, Xinxin Ding, Irvin S. Y. Chen, Yongping Jiang

**Affiliations:** 1 Biopharmaceutical R&D Center, Chinese Academy of Medical Sciences & Peking Union Medical College, Suzhou, China; 2 Biopharmagen Corp., Suzhou, China; 3 College of Life Science and Technology, Beijing Advanced Innovation Center for Soft Matter Science and Engineering, Beijing University of Chemical Technology, Beijing, 100029 China; 4 Division of Chemical Biology and Medicinal Chemistry, Eshelman School of Pharmacy and Carolina Center for Genome Sciences, University of North Carolina, Chapel Hill, NC USA; 5 Department of Pharmacology and Toxicology, College of Pharmacy, University of Arizona, Tucson, AZ US; 6 Department of Microbiology, Immunology, and Molecular Genetics, David Geffen School of Medicine at University of California, University of California Los Angeles, Los Angeles, CA USA

**Keywords:** Molecular biology, Drug delivery, Cancer therapy, RNA, Cancer

## Abstract

An *in vitro*-transcribed RNA aptamer (trans-RA16) that targets non-small cell lung cancer (NSCLC) was previously identified through *in vivo* SELEX. Trans-RA16 can specifically target and inhibit human NCI-H460 cells *in vitro* and xenograft tumors *in vivo*. Here, in a follow-up study, we obtained a chemically-synthesized version of this RNA aptamer (syn-RA16) and a truncated form, and compared them to trans-RA16 for abilities to target and inhibit NCI-H460 cells. The syn-RA16, preferred for drug development, was by design to differ from trans-RA16 in the extents of RNA modifications by biotin, which may affect RA16’s anti-tumor effects. We observed aptamer binding to NCI-H460 cells with K_D_ values of 24.75 ± 2.28 nM and 12.14 ± 1.46 nM for syn-RA16 and trans-RA16, respectively. Similar to trans-RA16, syn-RA16 was capable of inhibiting NCI-H460 cell viability in a dose-dependent manner. IC_50_ values were 118.4 nM (n = 4) for syn-RA16 and 105.7 nM (n = 4) for trans-RA16. Further studies using syn-RA16 demonstrated its internalization into NCI-H460 cells and inhibition of NCI-H460 cell growth. Moreover, *in vivo* imaging demonstrated the gradual accumulation of both syn-RA16 and trans-RA16 at the grafted tumor site, and qRT-PCR showed high retention of syn-RA16 in tumor tissues. In addition, a truncated fragment of trans-RA16 (S3) was identified, which exhibited binding affinity for NCI-H460 cells with a K_D_ value of 63.20 ± 0.91 nM and inhibited NCI-H460 cell growth by 39.32 ± 3.25% at 150 nM. These features of the syn-RA16 and S3 aptamers should facilitate the development of a novel diagnostic or treatment approach for NSCLC in clinical settings.

## Introduction

Lung cancer is the leading cause of cancer-related mortality in men and the second leading cause in women worldwide^1–3^. Thus far, the two main types of lung cancer are non-small cell lung cancer (NSCLC) and small cell lung cancer (SCLC). NSCLC accounts for 85–90% of all lung cancer cases^4^. Chemotherapy is the primary treatment of choice for NSCLC; however, there are side effects such as gastrointestinal distress, organ damage, and even death^5,6^. In comparison with SCLC, NSCLC is relatively insensitive to chemotherapy, thus increasing the risk of mortality. In order to increase the survival rate of patients with NSCLC, there has been great interest in developing new targeted therapies and combination therapies.

Recently, the specific targeting of monoclonal antibodies and Chimeric Antigen Receptor (CAR)-T cells have contributed to the treatment of cancers including NSCLC. However, the high cost and sophisticated techniques required for manufacturing these bio-products are major challenges in clinical applications^7–11^. Aptamers are a class of single-stranded oligonucleotides (RNAs or ssDNAs) that can serve as ligands that recognize and bind to their targets with specificity and high affinity^12^. Typically, specific aptamers are generated by a process known as Systematic Evolution of Ligands by Exponential Enrichment (SELEX)^13,14^. Since the invention of SELEX in the 1990s, specific aptamers for various targets have been identified^15–17^.

A previous study by our group demonstrated the potential of a NSCLC-specific RNA aptamer selected via *in vivo* SELEX^18^. The aptamer, named RA16, was capable of binding to and inhibiting NSCLC human large cell lung cancer cell line NCI-H460 cells *in vitro* and *in vivo*, which may be applied to tumor imaging technique and targeted therapies. A major advantage of RNA aptamers is that they can be chemically synthesized for use in diagnosis, treatment and biomarker discovery. Therefore, the binding and inhibitory activity of the synthesized RA16 (syn-RA16), as well as the potential mechanisms should be further investigated. Furthermore, a smaller aptamer size could facilitate large-scale chemical synthesis and would be beneficial for clinical applications.

Here, we conducted a sequential study of the syn-RA16 and truncated aptamers specifically targeted and directly inhibited towards NCI-H460 cells *in vitro* and *in vivo*. We also demonstrated the potential tentative mechanism for syn-RA16 internalization and intracellular signaling mechanism.

## Results

### Specificity and Affinity of syn-RA16 *in vitro*

**Figure 1:**
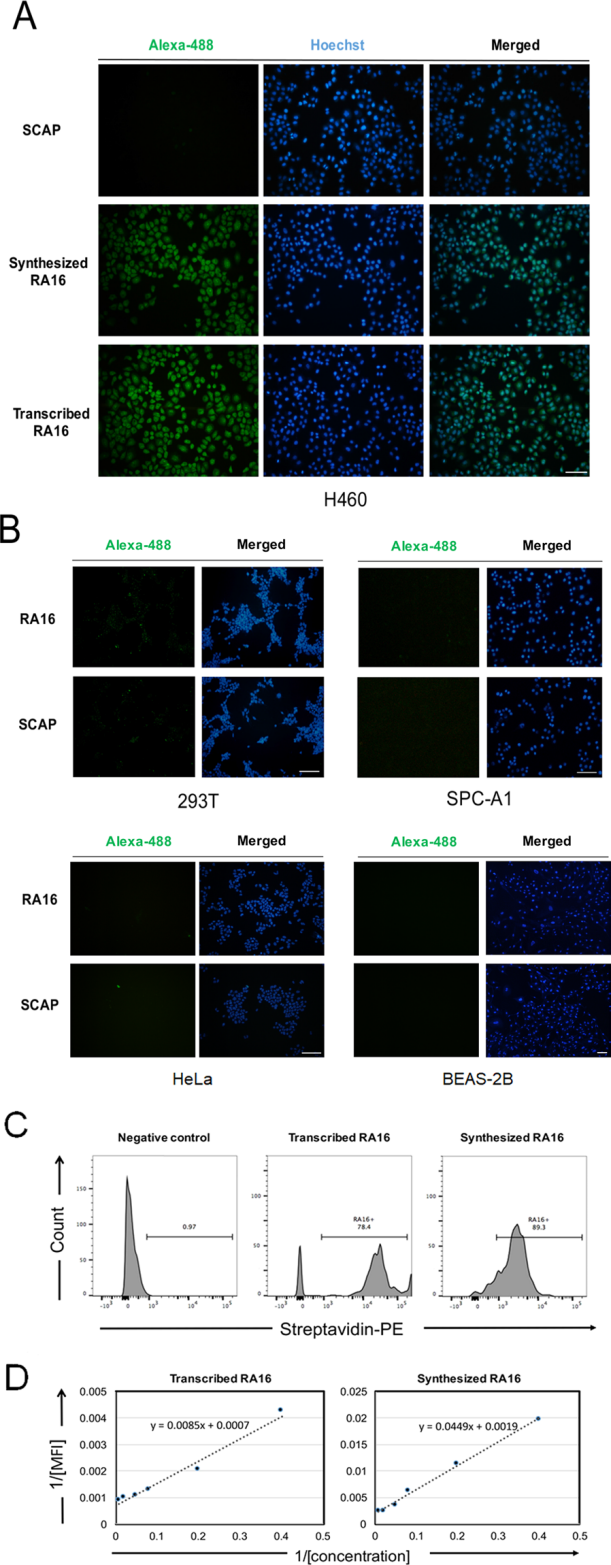
Specificity and Affinity of RA16 aptamers for NCI-H460 cells. (**A**) Representative fluorescence microscopy images showing the binding of RNA aptamers to NCI-H460 cells. NCI-H460 cells were incubated with biotin-labeled RNA molecules and detected using streptavidin-Alexa Fluor 488 (10× objective, bar = 100 μm). (**B**) Fluorescence microscopy analysis of the binding of RA16 aptamers to different cells. HEK-293T, SPC-A1, HeLa, and BEAS-2B cells were incubated with biotin-labeled RA16 or SCAP, and the images of the cells were obtained after 1 h of incubation using streptavidin-Alexa Fluor 488 (10 × objective, bar = 100 μm). Alexa-488 staining represents the aptamer, and merged represents the nucleus (Hoechst staining) and aptamers. (**C**) Flow cytometry analysis of the aptamer binding to NCI-H460 cells. NCI-H460 cells were incubated with biotin-labeled RNA molecules, and fluorescence intensities were detected by flow cytometry using streptavidin-PE. (**D**) The mean fluorescence intensity (MFI) of biotin-labeled syn-RA16 and trans-RA16 (5–200 nM) was determined, and binding affinity was determined by linear fitting.

We previously reported that the NSCLC-specific RA16 selected via *in vivo* SELEX could bind to NSCLC NCI-H460 cells *in vitro*. To further confirm the specificity of the selected syn-RA16 aptamer, its binding activity was evaluated and compared with that of trans-RA16 *in vitro*. The syn-RA16 or biotin-labeled trans-RA16 aptamer was incubated with NCI-H460 cells, NSCLC (human lung adenocarcinoma cell line SPC-A1 cells), or control cells including human embryonic kidney-293T (HEK-293T) cells, human cervical carcinoma cell line (HeLa cells), and human normal lung cell line (BEAS-2B cells). Scrambled RNA (SCAP) served as the negative control. Fluorescence was detected using streptavidin-Alexa Fluor 488, and the cells were imaged under a microscope. Fluorescent binding was observed for NCI-H460 cells, but not SPC-A1, HEK-293T, HeLa, and BEAS-2B cells (Fig. 1A,B), suggesting that syn-RA16, similar to trans-RA16, demonstrated specific binding to NCI-H460 cells *in vitro*.

To assess the binding affinity of syn-RA16 and compare it with trans-RA16, flow cytometry was performed. As shown in Fig. 1C, when NCI-H460 cells were incubated with biotin-labeled RNA molecules, syn-RA16, similar to trans-RA16, bound to most of the cells and exhibited a clear fluorescence shift detected by streptavidin-PE, thus indicating the specific binding between syn-RA16 and NCI-H460 cells. There was no noticeable fluorescence shift for the negative control (Fig. 1C). We then determined the binding affinity of syn-RA16 and trans-RA16 for NCI-H460 cells by measuring the equilibrium dissociation constant (K_D_). Analysis of the fitted lines revealed K_D_ values of 24.75 ± 2.28 nM and 12.14 ± 1.46 nM for syn-RA16 and trans-RA16, respectively (Fig. 1D), demonstrating the high affinity of the aptamers for NCI-H460 cells.

### Specific binding mediated internalization of aptamer RA16

As reported, most cell-binding aptamers rely on cell membrane biomarkers for recognition and internalization through endocytosis^19–21^. To further investigate the time course of RA16 and potential mechanism for aptamer internalized, we performed time course study to monitor aptamer syn-RA16 internalization with different incubation time points.

**Figure 2:**
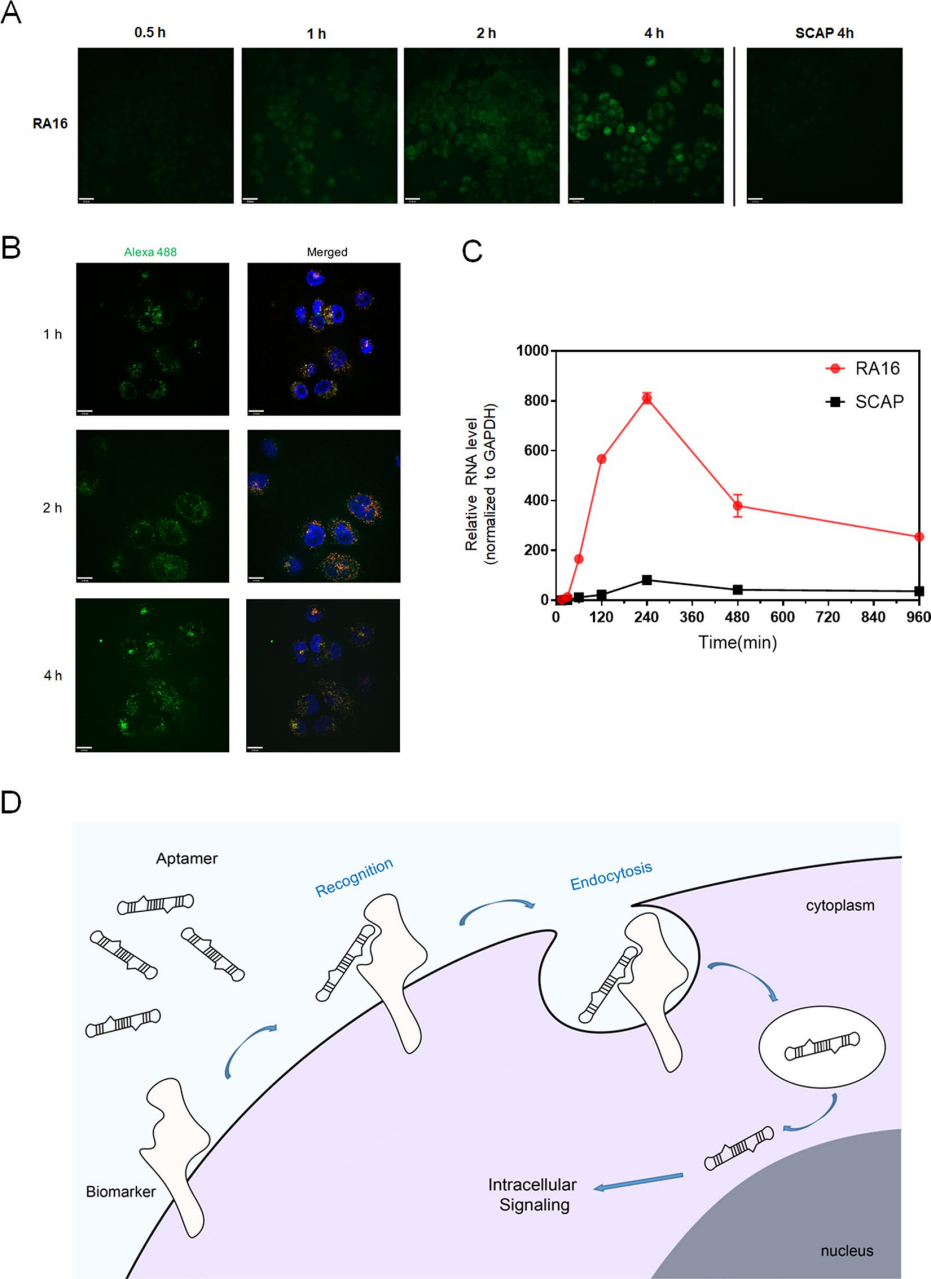
Tentative mechanism for RA16 aptamer internalization. (**A**) Time course for aptamer syn-RA16 entering NCI-H460 cells. After NCI-H460 cells were incubated with syn-RA16 for 0.5, 1, 2, and 4 hours, fluorescence signal of syn-RA16/SCAP was then detected using streptavidin-Alexa Fluor 488 (Scale bar = 35 μm). (**B**) Representative confocal microscopy images of NCI-H460 cells show significant colocalization between syn-RA16 and LysoTracker (endosome/lysosomes). Alexa-488 staining represents the aptamer, and merged staining represents aptamer(green), endosome/lysosomes(red) and nucleus(blue) localization (Scale bar = 10 μm). (**C**) Quantification of aptamer syn-RA16 entering NCI-H460 cells. After NCI-H460 cells were incubated with syn-RA16/SCAP for series time points, relative RNA levels in cells were analyzed by qRT-PCR (normalized to GAPDH). All data represent the mean ± SD, n = 4. (**D**) Mechanism of aptamer-mediated cell binding and intracellular signaling courses. The RA16 specifically bound the aptamer cell biomarkers and internalized in the cells. The aptamer finally accumulated in the cytoplasm, resulting in intracellular signaling pathway.

As shown in Fig. 2A, syn-RA16 gradually entered NCI-H460 cells, and binding of syn-RA16- NCI-H460 cells was detectable after 1 h incubation. When the incubation time was increased to 2 h, RA16 gradually entered the cell and signaled at the most in cytoplasm. Colocalization of aptamer RA16 and lysotracker revealed that the most of the aptamer entered cells through endocytosis (Fig. 2B). After 4 h incubation, the syn-RA16 further accumulated in the cytoplasm.

To further quantify time-depending internalization of RA16 to NCI-H460 cells, NCI-H460 cells were incubated with syn-RA16 or SCAP in serum containing medium for different incubation time. The relative aptamer recovery levels were further detected by quantitative RT-PCR. The internalized aptamer kinetics were shown in Fig. 2C. Consistent with the imaging data, SCAP showed low internalization in NCI-H460 cells. On the contrast, syn-RA16 demonstrated a significantly strong internalization. As time increasing, syn-RA16 gradually internalized and reached at the maximum after 4 h incubation.

The results indicated that aptamer RA16 entered NCI-H460 cells through receptor-mediated endocytosis. The specific binding triggered the aptamer internalized, migrated, and finally accumulated in the cytoplasm. The possible mechanism of aptamer binding and signaling courses is shown in Fig. 2D.

### Inhibition of cell growth *in vitro*

**Figure 3:**
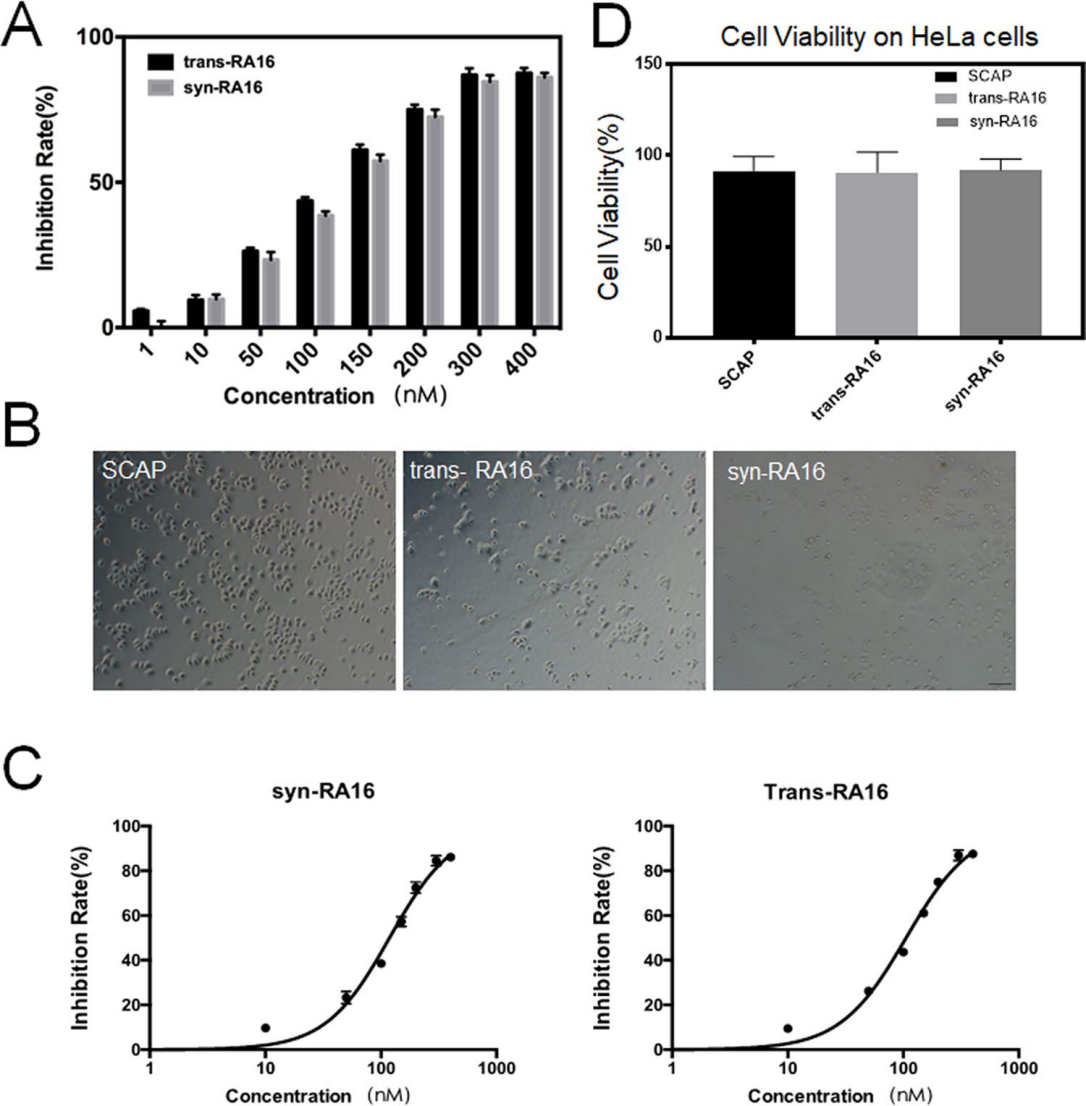
Inhibitory activity of RA16 aptamers towards NCI-H460 cells. (**A**) NCI-H460 cell viability was evaluated with a standard Cell Counting Kit-8 (CCK-8) assay after treatment with different doses of trans-RA16 or syn-RA16 for 48 h. All data represent the mean ± SD, n = 4. (**B**) Microscopy analysis of NCI-H460 cell apoptosis after treatment with RNA molecules. NCI-H460 cells were treated with SCAP, trans-RA16, or syn-RA16 and observed under the microscope (10× objective, bar = 100 μm). (**C**) IC_50_ curves of the inhibition of NCI-H460 cell growth by trans-RA16 or syn-RA16. All data represent the mean ± SD, n = 4. (D) HeLa cell viability was evaluated with a standard Cell Counting Kit-8 (CCK-8) assay after treatment with 600 nM SCAP, trans-RA16 or syn-RA16 for 48 h. All data represent the mean ± SD, n = 4.

Trans-RA16 has been demonstrated previously to inhibit NCI-H460 cell growth^18^. Therefore, we performed cell viability assays to assess the inhibitory activity of syn-RA16. As shown in Fig. 3A, the inhibition rates of NCI-H460 cell growth at various concentrations by syn-RA16 and trans-RA16 were almost similar. Notably, syn-RA16 suppressed NCI-H460 cells by 84.5%, whereas trans-RA16 suppressed the cells by 86.8% at 300 nM. NCI-H460 cells treated with both trans-RA16 and syn-RA16 exhibited an apoptotic phenotype (Fig. 3B). Furthermore, analysis of the inhibition rate with a concentration series of RA16 revealed IC_50_ values of 118.4 nM and 105.7 nM for syn-RA16 and trans-RA16, respectively (Fig. 3C). Interestingly, both syn-RA16 and trans-RA16 did not have an inhibitory effect on HeLa cells at 600 nM (Fig. 3D).

### Tumor-targeting efficacy *in vivo*

**Figure 4:**
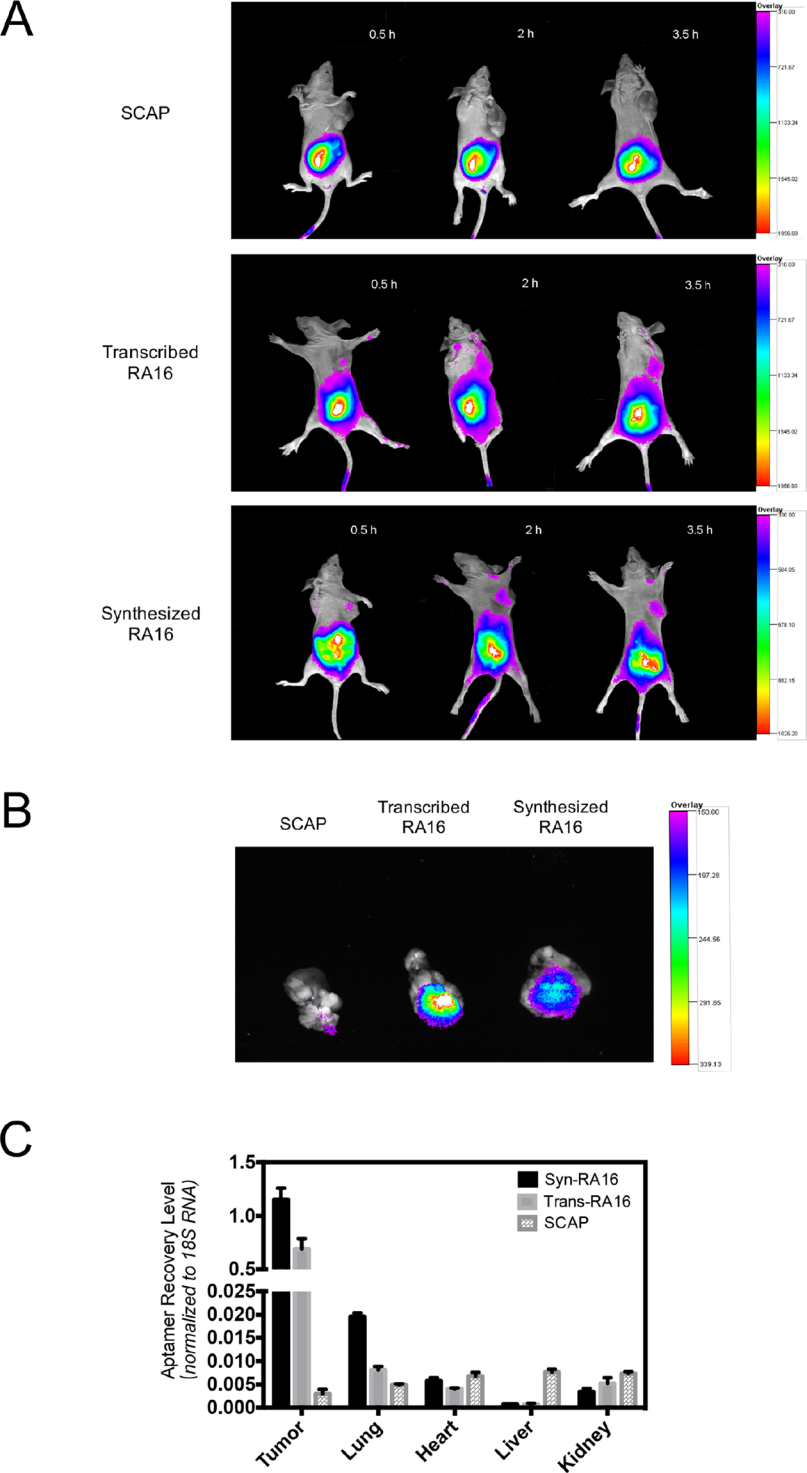
Tumor-specific targeting efficacy of RA16 aptamers *in vivo*. (**A**) *In vivo* tumor imaging of Cy5.5-labeled RNA molecules in NCI-H460 tumor-bearing mice at different time points (0.5, 2, and 3.5 h post-injection). (**B**) Imaging of the RNA molecules in tumors extracted from tumor-bearing mice after 4 h of circulation. (**C**) Relative RNA levels in tumor, lung, liver, heart, and kidney tissues analyzed by qRT-PCR (normalized to mouse 18S RNA). All data represent the mean ± SD, n = 4.

After evaluating the specificity and inhibitory activity of syn-RA16 *in vitro*, we further performed an *in vivo* tumor imaging assay to investigate the targeting activity of syn-RA16 *in vivo*. Cy5.5-labeled syn-RA16 or trans-RA16 was injected into NCI-H460 tumor-bearing mice to track the movements of the specific RA16 molecules *in vivo*. SCAP was used as the control. At 0.5 h, syn-RA16 and trans-RA16 reached the tumor site with a weak fluorescence signal. As time increased, RA16 aptamers gradually accumulated at the tumor site with a strong fluorescence signal at 2 h. Notably, no fluorescence signal was observed in the tumors of mice injected with SCAP (Fig. 4A). Subsequently, we extracted the tumors for imaging; fluorescence signals were much higher in mice injected with syn-RA16 or trans-RA16 than in those injected with SCAP (Fig. 4B), demonstrating the specific target binding of syn-RA16 *in vivo*.

An *in vivo* trap assay with quantitative reverse transcription polymerase chain reaction (qRT-PCR) was also performed to evaluate the distribution of syn-RA16 *in vivo*. NCI-H460 tumor-bearing mice were injected with syn-RA16, trans-RA16, or SCAP. Total RNA was then collected from various tissues for RNA quantification (normalized to mouse 18S RNA). The graph of RNA distribution is shown in Fig. 4C (n = 4). The level of syn-RA16 was significantly higher (50- to 1000-fold) in tumor tissues than in other tissues such as liver, kidney, heart, and lung tissues. Similar results were observed for trans-RA16. Moreover, the level of RNA molecules in the tumor tissues was significantly different between the RA16 groups and SCAP group. As shown in Fig. 4C, trapped RA16 was 100-fold higher than trapped SCAP at the tumor site, indicating that the entrapment of syn-RA16 in tumor tissues may be attributed to its specific binding *in vivo*. Overall, the results demonstrated the tumor-specific targeting activity of syn-RA16 and trans-RA16 both *in vitro* and *in vivo*.

### Cell binding and inhibitory activity of a truncated aptamer

**Figure 5:**
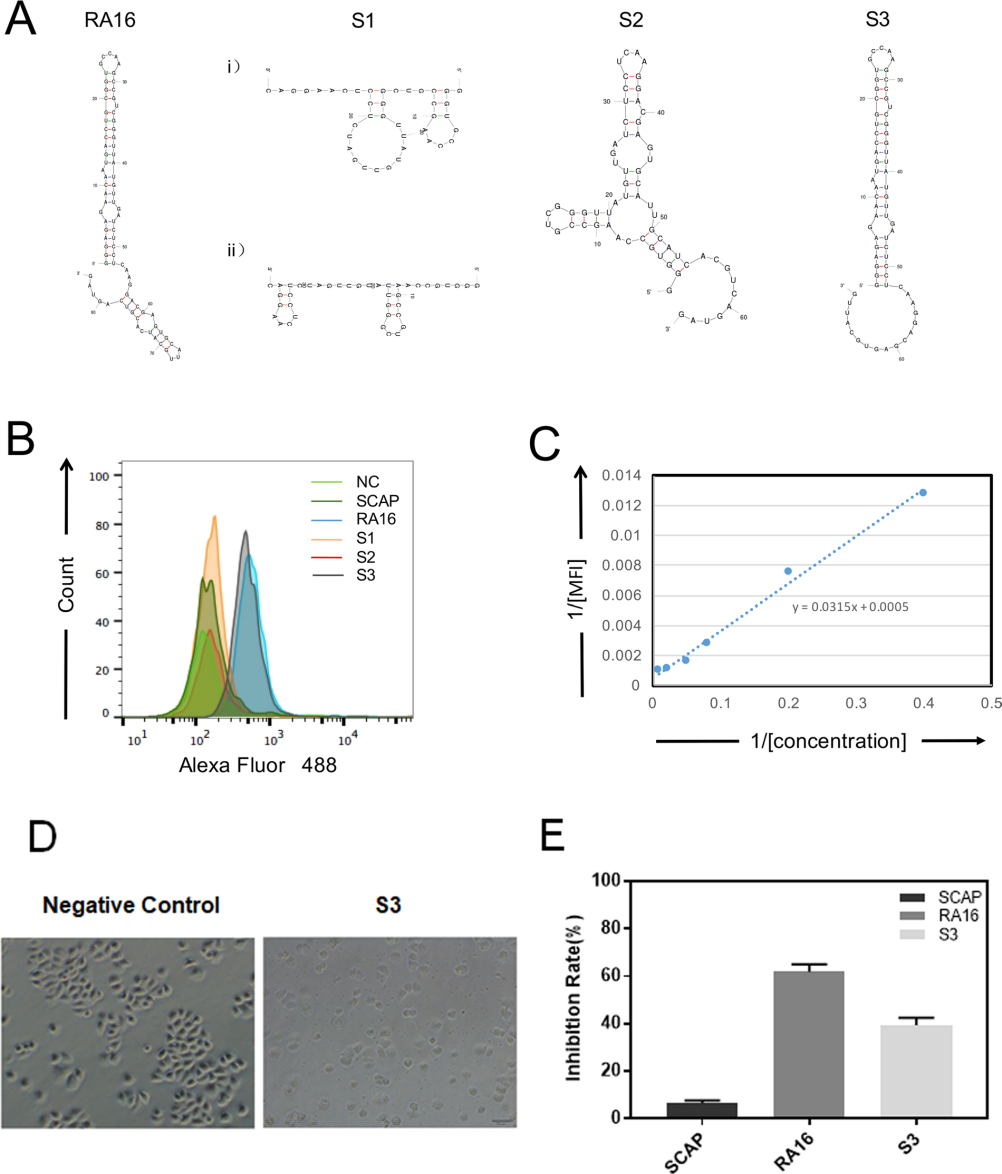
Binding and inhibitory activity of the truncated aptamer (S3). (**A**) Secondary structures of the truncated aptamer predicted by Mfold software (http://unafold.rna.albany.edu/?q=mfold). (**B**) Flow cytometry analysis of the truncated aptamer binding to NCI-H460 cells. NCI-H460 cells were incubated with biotin-labeled RNA fragments, and fluorescence intensities were detected by flow cytometry using Streptavidin-Alexa Fluor 488. (**C**) The mean fluorescence intensity (MFI) of biotin-labeled S3 (5–200 nM) was determined, and binding affinity was evaluated by linear fitting. (**D**) Microscopy analysis of NCI-H460 cell apoptosis after treatment with truncated aptamer. NCI-H460 cells were treated with 150 nM PBS or S3 and observed under the microscope (20× objective, bar = 50 μm). (**E**) NCI-H460 cell viabilities were evaluated with a standard CCK-8 assay after 48-hr incubation with 150 nM SCAP, RA16 or S3. All data represent means ± SD, n = 4.

In order to minimize the functional motif and facilitate large-scale chemical synthesis, RA16 aptamers were truncated into three smaller parts (S1 containing 40 random nucleotides, S2 containing 40 nucleotides with the 3′-end, and S3 containing the 5′-end with 40 random nucleotides). The potential secondary structures of RA16 and three smaller fragments were predicted by Mfold software (http://unafold.rna.albany.edu/?q=mfold), as shown in Fig. 5A^22^.

Binding activity was assessed by flow cytometry. As shown in Figs. 5B and S1, S2, and SCAP (negative control) were unable to bind to the target cells. However, S3, similar to RA16, exhibited binding affinity for NCI-H460 cells. The binding affinity (K_D_) was 63.20 ± 0.91 nM (Fig. 5C).

In addition, the inhibitory activity of S3 was further determined. As shown in Fig. 5D,E, similar to RA16, NCI-H460 cells treated with S3 exhibited an apoptotic phenotype. S3 inhibited H460 cell growth by 39.32 ± 3.25% at 150 nM, while RA16 suppressed the cells by 61.79 ± 3.27%. These results indicated that S3 containing the 5′-end with 40 random nucleotides of RA16 retained cell-binding and inhibitory activity.

## Discussion

NSCLC is one of the leading causes of cancer-related deaths worldwide^4^. Although there are severe side effects, traditional treatment strategies, such as chemotherapy, remain the primary treatment of choice in clinical settings^5,6^.

There have been various efforts to develop novel targeted therapeutics and overcome the drawbacks of chemotherapy. In comparison with chemotherapy, small molecules such as epidermal growth factor receptor (EGFR) tyrosine kinase inhibitors (TKIs) have been reported to play a major role in enhancing the survival rate of patients with NSCLC and decreasing toxicity^23^. However, studies have reported that EGFR TKIs did not affect patients harboring wild-type EGFR^6,24^. Furthermore, resistance can invariably occur^23,24^. Monoclonal antibodies such as cetuximab and bevacizumab may be effective in attenuating the progression of lung cancer^7,8,10^. However, they face similar resistance problems^23^. In comparison with tumor-targeting monoclonal antibodies, aptamers have several advantages such as (i) production via chemical synthesis, (ii) no or low immunogenicity, (iii) a smaller molecular size, (iv) efficient biological compartments penetration, and (v) ease of conjugation to various nanomaterials^25–28^. Previous studies have revealed that specific cancer aptamers are useful for *in vitro* tumor diagnosis, *in vivo* tumor imaging technique^29–32^, and targeted tumor therapy^2,20,33–35^.

Owing to their smaller size, specific binding, and tissue-penetration activity, RNA aptamers are considered as ideal agents for cancer diagnosis and cancer-targeted therapy. Aptamers specific for cancer-related proteins including vascular endothelial growth factor (VEGF), EGFR, mucin 1 (MUC1), and p53 have been identified^15,31,32,36^. Previous studies on targeted chemotherapeutic delivery and tumor imaging have demonstrated the potential of aptamers for targeted treatment and cancer diagnosis^29,30,33,37–40^. Recently, an NSCLC-specific RNA aptamer was selected via *in vivo* SELEX^18^. Binding activity of RA16 to NSCLC cell line (NCI-H1299, SPC-A1, and NCI-H1650 cells), as well as non-NSCLC (HeLa and 293 T cells) were detected respectively, which demonstrated high specificity and affinity towards specific NSCLC tumors. A major advantage of aptamers is the ease of chemical synthesis. Giving synthetic RNA aptamers have a more uniform and highly purified consistent stable structure, the syn-RA16 could easily be adopted for large-scale and cost-efficient production in clinical application. In addition, the syn-RA16 would be beneficial for further modifications such as incorporation of 2′-F dCTP/UTP and 5′-PEGylation, as well chemical adducting and manufacturing^18^. Obviously, the advantages of synthesized aptamers would be more feasible for applications of the clinic.

In this study, we evaluated the specific target binding and direct inhibitory activity of syn-RA16. As we tested and determined the binding affinity in the preliminary study, most of the non-NSCLC cell line showed no or little binding towards RA16, even at high concentration of syn-RA16 at 600 nM. It is our understanding that it’s impossible to determine the dissociation constant in lung normal cell lines and in non-NSCLC cell lines. We only determine the dissociation constant in NSCLC H460 cells. Although nucleotide sequences of syn-RA16 and transcribed RA16 are basically the same, syn-RA16 was produced by Dharmacon (GE Healthcare, Lafayette, CO), and trans-RA16 was transcribed from a DNA template *in vitro*. The main difference between syn-RA16 and trans-RA16 are their labeling status and purity. The affinity of syn-RA16 was slightly lower than that of trans-RA16 as demonstrated by K_D_ determination assay. This result may be attributed to the presence of only one biotin-labeled site in syn-RA16. On the other hand, additional biotin-labeled sites could be incorporated during the *in vitro* transcription process, resulting in a more sensitive fluorescence signal produced by trans-RA16. However, inhibitory activity was almost similar based on IC_50_ values for both syn-RA16 and trans-RA16 (118.4 nM vs. 105.7 nM). We also assessed the specific targeting of syn-RA16 by *in vivo* tumor imaging and qRT-PCR. Both syn-RA16 and trans-RA16 showed high retention in NCI-H460 tumor tissues *in vivo*. In fact, a more uniform flow binding profile with syn-RA16 was observed (Fig. 1C), indicating that the preparation of syn-RA16 was more purified than the trans-RA16. This result is consistent with a more tumor and lung bindings for syn-RA16 because with the same molar unit preparations of syn-RA16 and trans-RA16, the more active syn-RA16 aptamers were binding to tumor or lung tissues of NSCLC (Fig. 4C).

In addition, we further investigated the potential mechanism for aptamer binding and growth-inhibiting effects. Based on the time course and cell cycle analysis studies, it is our tentative hypothesis that aptamer RA16 firstly bound to the cell and triggered internalization, followed by further migration and accumulation in the cytoplasm. The internalized aptamer RA16 may regulate some intracellular pathways of NCI-H460 cells, such as, interfering the processes of the protein transcribing or translating in the cell cytoplasm. As a result, these effects may inhibit NCI-H460 cell growth. The tentative mechanism of the aptamer in cell binding and inhibition was proposed as shown in Fig. 2D. Moreover, our preliminary data (unpublished) showed that the target of RA16 is most likely a protein-related component.

On the other hand, a truncated fragment of RA16 (S3) was found to exhibit cell binding and inhibitory activity. Notably, more than three structures for S1 were predicted and only one possible structure for RA16 and S3 was predicted (as shown in Fig. 5A). S3 retained the secondary structure of RA16 at the 5′ end, and the other two truncated fragments (potentially folded into other structures) did not bind to target cells, indicating that the secondary/tertiary structure plays a major role in aptamer activity. The 5′-end structure of RA16 could be critical for specific binding and intracellular signaling. This region may induce aptamer internalization and lead to intracellular signaling for cell growth inhibition. However, the affinity (K_D_) and inhibitory effect of S3 were slightly lower than that of the full-length aptamer (63.20 ± 0.91 nM vs. 12.14 ± 1.46 nM; 39.32 ± 3.25% vs. 61.79 ± 3.27%) which is largely consistent with the truncation process, indicating that the 3′-end of the aptamer may also contribute to structure folding and target binding^41,42^.

In conclusion, we conducted a sequential study of the anti-NSCLC aptamer RA16, which can be chemically synthesized. In this study, syn-RA16 demonstrated specificity and high affinity for NSCLC NCI-H460 cells *in vitro* and *in vivo*. Notably, we demonstrated the tentative mechanism for syn-RA16 binding and intracellular signaling. In addition, the truncated aptamer (S3) exhibited cell binding and inhibitory activity, indicating that this aptamer could be further truncated and modified. The syn-RA16 and truncated aptamer could contribute to the identification of potential targets and elucidation of molecular mechanisms, which would be beneficial for future applications of the aptamer as a drug or diagnostic reagent for NSCLC.

## Materials and Methods

### Oligonucleotides

Full-length 2′-fluoropyrimidine RA16 aptamer (syn-RA16) was synthesized by Dharmacon (GE Healthcare, Lafayette, CO).

Syn-RA16: 5′-Biotin-GGGAGAGAACAAUGACCUGCGGUGCCAAGCCGUCGGGUUAUGUUGAUCUCCACAAGGACGAGUGCAUUGCAUCACGUCAGUAG-NH_2_-3′.

The DNA template for transcription and primers were synthesized by Integrated DNA Technologies (IDT, Coralville, IA).

DNA template: 5′-CACTAATACGACTCACTATAGGGAGAGAACAATGACCT GCGGTGCCAAGCCGTCGGGTTATGTTGATCTCCTCAAGGACGAGTGCATTGCATCACGTCAGTAG-3′. Forward primer: 5′-CACTAATACGACTCACTATAGGGAGAGAACAATG-3′. Reverse primer: 5′-CTACTGACGTGATGCAATGCACTC-3′.

### Cells

NCI-H460, HEK-293T, SPC-A1, HeLa, BEAS-2B, and other cell lines were purchased from the American Type Culture Collection (Manassas, VA). Tumor cell lines were cultured in Roswell Park Memorial Institute 1640 medium (Thermo Fisher Scientific, Rockford, IL). HEK-293T, HeLa, and BEAS-2B cells were cultured in Dulbecco’s modified Eagle’s medium (Thermo Fisher Scientific, Rockford, IL) containing 10% (v/v) fetal bovine serum, GlutaMax, and 100 U/mL penicillin-streptomycin in an incubator (Thermo Fisher Scientific, Rockford, IL) at 37 °C, 5% CO_2_. Cells were sub-cultured approximately every 2 days at 80% confluence using 0.25% (w/v) trypsin (Thermo Fisher Scientific, Rockford, IL) at a split ratio of 1:3.

### Animals

Female BALB/c nude mice were purchased from SLRC Laboratory Animal Center (Shanghai, China). All animal studies were performed in accordance with the Guide for Care and Use of Laboratory Animals, Soochow University. All animal experimental protocols were approved by the Soochow University Institutional Animal Care and Use Committee (IACUC Permit Number SYXK (Su) 2015-0105). The nude mice were injected with 2 × 10^6^ NCI-H460 cells subcutaneously to establish a tumor-bearing mouse model.

### Biotin labeling of transcribed aptamers

The DNA template of RA16 aptamers was *in vitro* transcribed into RNA in a reaction mixture consisting of 10× transcription buffer (400 mM Tris-Cl, 80 mM MgCl_2_, and 20 mM spermidine), 10 mM dithiothreitol, 20 U T7 mutant (Y639F) RNA polymerase, 10 mM ATP, 10 mM GTP (Sangon Technologies, Shanghai, China), 10 mM 2′-F-dCTP/UTP (TriLink Biotechnologies, San Diego, CA), 2 mM 16-Biotin-UTP (Sigma-Aldrich, St. Louis, MO), 20 U RiboLock RNase Inhibitor (Thermo Fisher Scientific, Rockford, IL), and 0.05 U inorganic pyrophosphatase (Thermo Fisher Scientific, Rockford, IL). The resulting reaction mixture was treated with 2 μL DNase I (5 U/μL, RNase-free; TaKaRa, Dalian, China) at 37 °C for 1 h, followed by phenol-chloroform extraction. RNA pellets were suspended in RNA refolding buffer (10 mM HEPES pH 7.4, 50 mM NaCl, 1 mM CaCl_2_,1 mM MgCl_2_, and 2.7 mM KCl), followed by refolding at 90 °C for 3 min and slowly cooling to room temperature^20^.

### Fluorescent labeling of aptamers

The DNA template was transcribed by substituting 16-Biotin-UTP for aminoallyl-dUTP (TriLink Biotechnologies, San Diego, CA) to generate aminated RNA. Both trans-RA16 and syn-RA16 were suspended in 0.1 M NaHCO_3_ (pH 8.3) and incubated with NHS-Cy5.5 (GE Healthcare, Marlborough, MA)^43^. After 2 h of reaction at room temperature, 10 mM Tris was added to neutralize excess fluorescent dye. Then, the mixture was filtered using Amicon YM-10 filter (Merck Millipore, Darmstadt, Germany) to generate fluorescently labeled RA16.

### Cell binding assay

NCI-H460, HEK-293T, SPC-A1, HeLa, and BEAS-2B cells were grown to 70% confluence in 24-well plates. After washing with Dulbecco’s phosphate-buffered saline (DPBS; Thermo Fisher Scientific, Rockford, IL) twice, the cells were incubated with 200 nM biotin-labeled aptamers in binding buffer (RNA refolding buffer containing 1% bovine serum albumin and 1.0 μg/mL tRNA) for 1 h at 37 °C^36^. Next, the cells were washed with RNA refolding buffer and stained with streptavidin-Alexa Fluor 488 (Thermo Fisher Scientific, Rockford, IL) according to the manufacturer’s protocol. The nucleus was stained with Hoechst 33342 (Thermo Fisher Scientific, Rockford, IL) at 37 °C for 5 min and washed with DPBS twice. Then, the cells were imaged under a microscope (Olympus, Tokyo, Japan).

### Affinity determination

NCI-H460 cells were digested with 0.25% trypsin. Next, 1 × 10^5^ cells were incubated with a concentration series (0 to 200 nM) of biotin-labeled syn-RA16 or trans-RA16 in 100 μL binding buffer for 1 h at 37 °C. The cells were then washed with DPBS twice and stained with streptavidin-PE (BioLegend, San Diego, CA) according to the manufacturer’s protocol^44^. After washing and re-suspension in fluorescence-activated cell sorting (FACS) buffer (BD Biosciences, San Diego, CA), the cells were subjected to flow cytometry analysis. A total of 10,000 events were acquired for each sample using the FACSVerse^®^ system (Becton Dickinson, Franklin Lakes, NJ), and data were obtained and analyzed by FlowJo^®^ software (version X 10.0, https://www.flowjo.com/). The mean fluorescence intensity (MFI) of each sample was determined to calculate the dissociation constant (K_D_) between aptamers and NCI-H460 cells by linear fitting according to the equation 1/F = (K_D_ + [L])/B_max_ × [L] (where F = fluorescence intensity and [L] = concentration of aptamers)^44,45^.

### Time course study

NCI-H460 cells were grown to 70% confluence on coverslips. The coverslips were washed with DPBS twice, and incubated with 200 nM biotin-labeled aptamers in binding buffer for 0.5 h, 1 h, 2 h, and 4 h at 37 °C. For the cells of colocalization, 100 nM LysoTracker™ Deep Red (Thermo Fisher Scientific, Rockford, IL) was added to the culture medium for imaging study. The coverslips were then fixed with 4% paraformaldehyde for 10 min and washed twice with DPBS. Cells were then stained with streptavidin-Alexa Fluor 488 according to the manufacturer’s protocol. The nucleus was stained with Hoechst 33342 at 37 °C for 5 min and washed with DPBS twice. The cells were then mounted and imaged under a confocal microscope (Olympus, Tokyo, Japan).

### Time course study with qRT-PCR

NCI-H460 cells were seeded in 24-well plates at 1 × 10^5^ cells per well overnight at 37 °C. The medium was then removed, and the cells were treated with 300 nM syn-RA16 or scrambled RNA (SCAP) in fresh medium for series incubation time (0, 15, 30, 60, 120, 240, 480, and 960 min). After incubation, cells were rinsed three times with DPBS, and then collected for RNA extraction using TRIzol reagent (Thermo Fisher Scientific, Rockford, IL) according to the manufacturer’s protocol. The extracted total RNAs were first treated with DNase I to eliminate DNA contamination and quantified using One Drop Spectrophotometry (Hong Kong, China). Next, 500 ng of DNase I-treated RNA was reverse-transcribed into DNA using M-MLV transcriptase (TaKaRa, Dalian, China). Real time PCR was performed with aptamer primers and Power SYBR Green Master Mix (Life Technologies) according to the manufacturer’s protocol, and the housekeeping gene glyceraldehyde-3-phosphate dehydrogenase (GAPDH, primers sets from Sangon Technologies, Shanghai, China) was amplified for normalization. Quantitative PCR data were analyzed using the StepOnePlus™ Real-Time PCR system (Applied Biosystems). The relative RNA levels with different incubation time were calculated by the 2^−ΔΔCT^ method using GAPDH as a control.

### Cell viability assay

NCI-H460 or other cells were seeded in 96-well plates at 5 × 10^3^ cells per well overnight. The medium was then removed, and the cells were treated with RNA molecules in fresh medium at different concentrations. After 48 h of incubation, cell viability was determined using Cell Counting Kit-8 (CCK-8; Dojindo, Tokyo, Japan). The absorbance was measured at 450 nm using a microplate reader (Thermo Fisher Scientific, Rockford, IL).

### *In vivo* imaging analysis

After the tumor grew to 200~300 mm^3^, tumor-bearing mice were administered with Cy5.5-labeled RNA molecules by tail vein injection. Cy5.5-labeled RNA molecules were tracked using an *in vivo* imaging system (Kodak FX Pro; Carestream Health, Rochester, NY) at 0.5, 2, and 3.5 h post-injection^18^. Tumors were then exacted for imaging after 4 h of circulation.

### *In vivo* trap assay with qRT-PCR

Three mice were administered with 1 nmol syn-RA16 or trans-RA16 via intravenous injection. After 3.5 h of circulation, tumor, heart, liver, lung, and kidney tissues were collected for RNA extraction using TRIzol reagent (Thermo Fisher Scientific, Rockford, IL) according to the manufacturer’s protocol. The resulting total RNA was first treated with DNase I to eliminate DNA contamination and quantified using One Drop (Hong Kong, China). Next, 500 ng of DNase I-treated RNA was reverse-transcribed into DNA using M-MLV transcriptase (TaKaRa, Dalian, China). qPCR was performed with RA16 aptamer primers and Power SYBR Green Master Mix (Life Technologies) according to the manufacturer’s protocol, and mouse 18S RNA (primers sets from Sangon Technologies, Shanghai, China) was amplified for normalization^18^. qPCR data were analyzed using the StepOnePlus™ Real-Time PCR system (Applied Biosystems). The relative RNA levels in various tissues were calculated by the 2^−ΔΔCT^ method using mouse 18S RNA as the control^46,47^.

### Truncation of RA16 aptamers

RA16 aptamers were truncated into three parts.

S1: 5′-GGGUGCCAAGCCGUCGGGUUAUGUUGAUCUCCUCAAGGAC-3′. S2: 5′-GGGUGCCAAGCCGUCGGGUUAUGUUGAUCUCCUCAAGGACGAGUGCAUUGCAUCACGUCAGUAG-3′ S3: 5′-GGGAGAGAACAAUGACCUGCGGUGCCAAGCCGUCGGGUUAUGUUGAUCUCCUCAAGGACGAGUGCAUUG-3′.

S1 was transcribed from the DNA sequence amplified by PCR using 5′-CACTAATACGACTCACTATAGGGTGCCAAGCCGTCGGGTTATGTTGATCTCCTCAAGGACGAGTGCATTGCATCACGTCAGTAG-3′ as template and the underlined sequences as primers.

S2 was transcribed from the DNA sequence amplified by PCR using 5′-CACTAATACGACTCACTATAGGGTGCCAAGCCGTCGGGTTATGTTGATCTCCTCAAGGACGAGTGCATTGCATCACGTCAGTAG-3′ as template and the underlined sequences as primers.

S3 was transcribed from the DNA sequence amplified by PCR using 5′-CACTAATACGACTCACTATAGGGAGAGAACAATGACCTGCGGTGCCAAGCCGTCGGGTTATGTTGATCTCCACAAGGACGAGTGCATTGCATCACGTCAGTAG-3′ as template and the underlined sequences as primers.

The *in vitro* transcription process was similar to that described for the full-length aptamer with the incorporation of 16-biotin-UTP. The secondary structures of the truncated aptamers, which include the possible binding region, were predicted by Mfold software (http://unafold.rna.albany.edu/?q=mfold).

### Statistical analysis

Results are presented as the mean ± standard deviation of at least three independent experiments with duplicate samples. Statistical differences were evaluated using one-way analysis of variance unless otherwise indicated. P < 0.05 was considered as statistically significant. Graphs were generated by GraphPad Prism (version 6; GraphPad, La Jolla, CA, USA) and Microsoft Excel (version 2010).
